# Case of Spontaneous Removal of a Cataract

**Published:** 1844-09

**Authors:** 


*Case of spontaneous Removal of a Cataract.*—Mr. R., a carpenter and joiner, ætat. 57, had cataract of the right eye fifteen years, and for five years has not been able to see at all with this eye. A short time ago, he consulted a well-known oculist in New York, who advised an operation, which circumstances obliged him to defer. About six weeks ago, while on a visit to a friend, he took up a pair of double convex spectacles, and applying them to his blind eye, discovered to his astonishment that he could see and even read small print. He consulted a surgeon on this singular occurrence, who informed him, that by some means which he could not account for, the cataract had become detached, leaving the pupil clear.

The eye now presents the following appearances. When at rest, the pupil is perfectly clear, and the contractions of the iris natural. The posterior chamber is very large, and the iris slightly tremulous. When the ball of the eye is moved, there suddenly shoots up behind the iris an opaque lens, of grayish color, medium size, and perfectly circular. It sometimes rises so high as to close the pupil entirely, particularly if the head is inclined forwards; generally it covers only the lower half of the pupil, and its motions are so rapid as not to interfere with vision any more than the act of winking. There is no pain in the eye, nor has there been any sign of inflammation. The patient is of very temperate habits, and is positive that he has never received a blow on the eye or head. He now uses this eye principally, on account of a cataract forming in the left eye.

From the size of the posterior chamber and the tremulous motion of the iris, I was at first inclined to account for the displacement of the lens by dissolution of the vitreous humor. But the globe of the eye is firm, shining, and elastic, the sclerotic of its natural color, and the sight good. Mereover, the vacillating motion of the iris is no greater than we often see after the removal of a cataract. In the four weeks that have elapsed since I first saw this case, I cannot detect any change in the appearance of the lens; it is probable that it is still enclosed in its capsule, which protects it from the dissolving properties of the aqueous humor.

This case is interesting, because it proves that cataract may sometimes be cured spontaneously. By a sudden jerk of the head, a fall, a blow on the eye or the temple, the opaque lens may be torn away from its natural connections, and removed entirely out of the axis of vision, leaving the eye in the same condition as after the operation by depression. Whether a change has taken place in the ciliary body, by which the connections of the lens are softened and loosened, is a question which we are not competent to answer. It is probable that some such change has occurred, or the spontaneous removal of the cataract would be of more frequent occurrence. Whatever this change may be, the present case shows that it is not always of sufficient gravity to interfere with perfect vision.

As long as the opaque lens remains behind the iris, it gives no uneasiness; but if it should pass into the anterior chamber, it is liable to create so much pain and inflammation, as to require its removal. This may be done by incision of the cornea, as in the operation of extraction, or we may adopt the expedient of Demours, and attempt to return it to the posterior chamber, by laying the patient on his back, and dilating the pupil largely with belladonna.—*Dr. Eustis, in N. O. Med. Jour.*

